# Quantitative ultrasound imaging of soft biological tissues: a primer for radiologists and medical physicists

**DOI:** 10.1186/s13244-021-01071-w

**Published:** 2021-09-09

**Authors:** Guy Cloutier, François Destrempes, François Yu, An Tang

**Affiliations:** 1 Laboratory of Biorheology and Medical Ultrasonics, Centre de recherche du Centre hospitalier de l’Université de Montréal (CRCHUM), 900 St-Denis, Montréal, Québec H2X 0A9 Canada; 2 Department of Radiology, Radio-oncology, and Nuclear Medicine, Université de Montréal, Montréal, Québec Canada; 3 Institute of Biomedical Engineering, Université de Montréal, Montréal, Québec Canada; 4 Microbubble Theranostics Laboratory, CRCHUM, Montréal, Québec Canada; 5 Department of Radiology, Centre hospitalier de l’Université de Montréal (CHUM), Montréal, Québec Canada; 6 Laboratory of Medical Image Analysis, Montréal, CRCHUM Canada

**Keywords:** Quantitative ultrasound imaging, Speed of sound imaging, Ultrasound attenuation imaging, Backscatter imaging, Backscatter coefficient

## Abstract

Quantitative ultrasound (QUS) aims at quantifying interactions between ultrasound and biological tissues. QUS techniques extract fundamental physical properties of tissues based on interactions between ultrasound waves and tissue microstructure. These techniques provide quantitative information on sub-resolution properties that are not visible on grayscale (B-mode) imaging. Quantitative data may be represented either as a global measurement or as parametric maps overlaid on B-mode images. Recently, major ultrasound manufacturers have released speed of sound, attenuation, and backscatter packages for tissue characterization and imaging. Established and emerging clinical applications are currently limited and include liver fibrosis staging, liver steatosis grading, and breast cancer characterization. On the other hand, most biological tissues have been studied using experimental QUS methods, and quantitative datasets are available in the literature. This educational review addresses the general topic of biological soft tissue characterization using QUS, with a focus on disseminating technical concepts for clinicians and specialized QUS materials for medical physicists. Advanced but simplified technical descriptions are also provided in separate subsections identified as such. To understand QUS methods, this article reviews types of ultrasound waves, basic concepts of ultrasound wave propagation, ultrasound image formation, point spread function, constructive and destructive wave interferences, radiofrequency data processing, and a summary of different imaging modes. For each major QUS technique, topics include: concept, illustrations, clinical examples, pitfalls, and future directions.

## Key points

- Quantitative ultrasound (QUS) provides images on interactions between ultrasound waves and biological tissues.
- Tissue-specific speed of sound images can be produced for QUS tissue characterization.
- Acoustic attenuation is used in QUS as biomarkers to produce images independent of ultrasound system characteristics and settings.
- Backscatter coefficient is independent of ultrasound system characteristics and settings for tissue characterization.

## Introduction

The field of quantitative ultrasound (QUS) imaging has been active for more than 50 years and it is only recently that ultrasound manufacturers have started implementing some of these biomarkers on clinical scanners. Several technical textbooks have described state-of-the-art innovations [1–4]. Interested readers may refer to a recent contribution for a thorough introduction to the field of QUS imaging [4]. Technical constraints such as the need to perform an additional acquisition on a reference phantom and measurement variability have delayed clinical adoption [5]. However, the development of dedicated instruments using QUS methods for the assessment of bone structures [6] and liver steatosis [7] has led to a resurgence of the field of QUS. Recently, major ultrasound radiology manufacturers have released speed of sound, attenuation, and backscatter packages for tissue characterization and imaging.

**Fig. 1:**
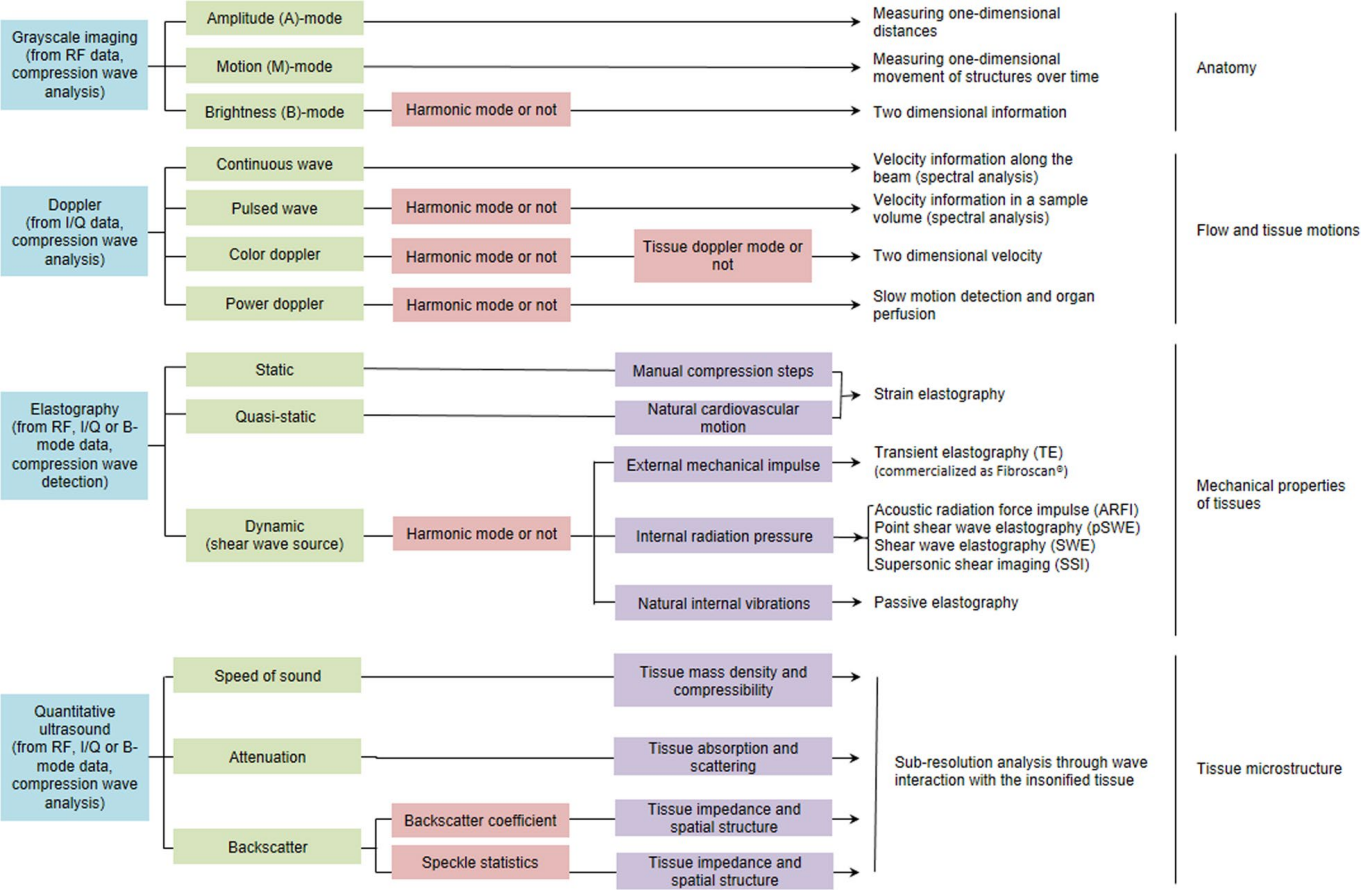
Classification of ultrasound techniques. From top to bottom, grayscale imaging modes provide information on organ anatomy. Doppler techniques assess flow and tissue motions (direction, velocity, and backscatter in power Doppler mode). Elastography methods provide information on mechanical properties of tissues. Quantitative ultrasound (QUS) detects sub-resolution acoustic properties to provide information on tissue microstructure. RF refers to radiofrequency data, I/Q to in-phase and quadrature data, whereas compression and shear waves refer to elastic waves used in ultrasound imaging

In contrast to brightness (B-mode) grayscale ultrasound imaging that provides qualitative information on anatomy, QUS aims at quantifying physical phenomena associated with the propagation of ultrasound into biological tissues. More specifically, QUS extracts fundamental properties of a tissue based on the interactions of propagating ultrasound waves with the tissue microstructure. These ultrasound sub-resolution quantitative signatures of the tissue microstructure are then used to produce a measurement of a global physical quantity within a region of interest (ROI) or parametric images for diagnosis. As illustrated in Fig. 1, these images are complementary to grayscale imaging, Doppler approaches measuring flow and tissue motion, and elastography (strain and shear wave based) assessing mechanical properties of tissues. While grayscale imaging, Doppler imaging, and elastography can also provide quantitative measures, the use of the terminology “quantitative” in QUS refers to a specific field dedicated to biomarkers describing wave interactions with the insonified organ.

The field of QUS imaging was initially described as “ultrasound tissue characterization (UTC)” [8]. Following the first international seminar on tissue characterization by ultrasound held in 1975 at the National Bureau of Standards in Washington, UTC was enthusiastically identified “to be one of the next big developments in the clinical utilization of ultrasound” (see this Editorial statement in [8]). After several decades of research marked by an unsuccessful translation of UTC technologies to ultrasound manufacturers and clinical practice, technical scientists strategically started introducing the concept of QUS, which in fact is in line with UTC initial developments aiming at providing biomarkers based on the interactions of ultrasound waves with the underlying tissue. QUS was first defined as a specific research field to characterize bone structures using attenuation and speed of sound measurements [9, 10]. UTC imaging is still used today to define this field of research [11], but it is no longer specific to QUS technologies based on the physics of wave interactions with biological tissues [12]. Indeed, UTC also refers to image processing methods to extract tissue information.

In this article, we refer to QUS measurements or images with a focus on soft tissue characterization, and on examples of QUS methods applied to grade liver steatosis and breast cancer (because those applications are the first ones using implementations of QUS on clinical scanners). Basic concepts behind speed of sound, attenuation, and backscatter measurements to grade a normal or a pathological state of an organ are reviewed. We describe key QUS technical concepts and illustrate QUS methods by avoiding complex mathematical equations. We provide a glossary of key terms relevant to this field (Table 1). To understand QUS methods, this article reviews basic concepts on the type of acoustic waves, speed of sound of these waves, ultrasound wave propagation phenomena, image formation, point spread function, constructive and destructive wave interferences, and radiofrequency data processing versus B-mode images. For each QUS method, topics include simplified concepts, units, range of values for soft biological tissues, measurement methods, illustrations, clinical examples, pitfalls, and future directions. For a few technical descriptions, the label “*advanced materials*” is used and the text is in italic.

**Table 1:** Glossary of commonly used terms in quantitative ultrasound

Term	Definition
Absorption	Loss in energy of the propagating compression wave due to local tissue heating
Acoustic impedance	Product of the speed of sound by the density of the tissue
Attenuation	Decrease in amplitude of acoustic waves propagating through soft tissues; caused by loss of mechanical energy due to wave absorption, reflection, refraction, diffraction, and scattering
Attenuation coefficient	Ratio of one radiofrequency echo magnitude to another at a different depth; expressed in decibel per centimeter per megahertz (dB/cm/MHz)
Attenuation coefficient slope	Linear relation between the attenuation coefficient and frequency; expressed in dB/cm
Backscatter	Analysis of echoes received by the transducer due to reflection and scattering of compression waves
Backscatter coefficient (BSC)	Formal definition of the backscatter intensity returned by a tissue and defining its sub-resolution structure; expressed in cm/steradian
Brightness mode (B-mode)	Ultrasound mode providing two-dimensional images in grayscale for assessment of anatomy
Compression wave	Type of acoustic wave in which the oscillation motion is parallel to the direction of wave propagation; also known as a longitudinal wave
Diffraction	Type of interaction between a wave and a physical medium in which the sound is dispersed when travelling through a hole smaller than the wavelength
Homodyned-K (HDK) statistical models	Descriptive statistical model used to fit the histogram distribution of ultrasound speckle with 3 parameters for tissue characterization
In-phase and quadrature (I/Q) demodulated data	Low-frequency representation of the radiofrequency signal obtained by the quadrature demodulation process
Nakagami statistical models	Descriptive statistical model used to fit the histogram distribution of ultrasound speckle with 2 parameters for tissue characterization
Point spread function (PSF)	Response of an ultrasound system to a single reflector much smaller than the acoustic wavelength but with sufficient impedance to generate an echo
Quantitative ultrasound (QUS)	Field of ultrasound imaging that aims to quantify the interactions between a compression acoustic wave and a biological tissue for its structural sub-resolution characterization
Radiofrequency (RF)	Acoustic signals detected by the ultrasound transducer with a frequency bandwidth dictated by the ultrasound probe characteristics
Reflection	Type of interaction between an acoustic wave and a physical medium in which the wave bounces back at the same angle but at a different direction; reflections generate an echo detected by the ultrasound transducer when transmitted and reflected angles are in the field of view of the probe
Refraction	Type of interaction between an acoustic wave and a physical medium in which the wave is bent at an angle and travels at a different speed due to a mismatch in acoustic impedance of encountered tissue interfaces
Scattering	Type of interaction between an acoustic wave and a physical medium in which the wave in bounces at angles of 360 degrees; scattering occurs when the tissue structure is much smaller than the acoustic wavelength
Shear wave	Type of acoustic wave in which the oscillation motion is perpendicular to the direction of the wave propagation; also known as transverse waves
Speed of sound (SoS)	Square root of the bulk elasticity modulus of the tissue divided by its density; expressed in m/s
Structure factor size estimator (SFSE)	Spectral representation of the backscatter coefficient modeled with 2 fitting parameters obtained by considering wave interference phenomena with a structure factor term, from which is extracted the packing factor and the mean size of scatterers
Ultrasound tissue characterization (UTC)	Historical term to describe the field of quantitative ultrasound; nowadays, the term QUS is preferred because UTC is also used to describe image processing strategies to extract image characteristics of a tissue

## Background on ultrasound wave propagation and image processing

### Types of acoustic waves

**Fig. 2:**
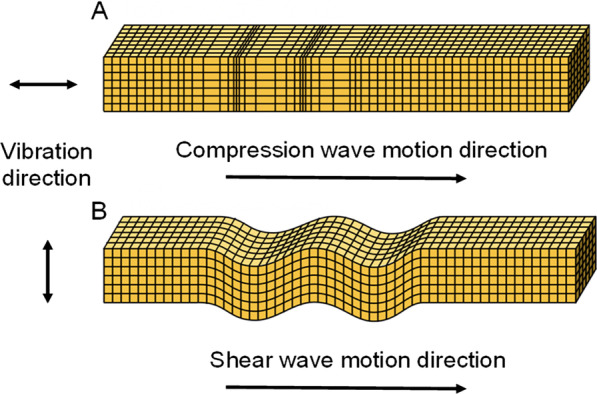
Two types of mechanical waves are used in ultrasound imaging. **a** Compression waves, also known as longitudinal waves, consist in alternating compressions and dilations of the insonified tissue with the moving wave direction parallel to the direction of the source. As indicated in Fig. 1, compression waves are used in all imaging modes (grayscale, Doppler, strain and shear wave elastography, and QUS). **b** Shear waves, also known as transverse waves, consist in alternating shearing motion of the insonified tissue with the moving wave direction perpendicular to the direction of the source. This type of wave is used in shear wave elastography to produce the source of motion, whereas compression waves are used for detection and imaging for all imaging modes, including detection of the shear wave in shear wave elastography (see Fig. 1)

In fundamental acoustics, different types of mechanical waves, called elastic waves, can propagate into soft biological tissues. Among those, compression and shear waves are currently used on clinical ultrasound scanners (Fig. 2). Compression waves, also known as longitudinal waves, consist in alternating compressions and dilations of the tissue, where the direction of wave propagation is parallel to the direction of the source. Compression waves are used in all imaging modes (grayscale, Doppler, elastography, and QUS). Shear waves, also known as transverse waves, consist in alternating shearing of the tissue, where the oscillation motion is perpendicular to the direction of the wave propagation. Besides compression and shear waves, other types of elastic waves can travel into biological tissues as surface waves (i.e., Rayleigh and Love waves) [13, 14]. The latter types of waves are being investigated for ultrasound imaging research and developments [15, 16].

### Speed of sound

The speed of sound of compression waves is fixed on most ultrasound scanners to 1,540 m/s, and this value is used to convert time to distance for producing an image. Indeed, ultrasound systems are measuring echo times between emission and reception, and a fixed speed of sound is assumed to map received echoes into spatial depth. In reality, the actual speed of sound varies depending on tissue structural characteristics. This assumption produces errors as the real speed of sound in soft tissues varies by ± 150 m/s or about 10% of the assumed speed of sound [17, 18]. In QUS, tissue-dependent variations in speed of sound are retrieved and used as a biomarker of a pathological state. Shear waves are much slower and travel at a speed of a few m/s in biological tissues (typically < 10 m/s). In shear wave elastography, the shear wave speed is used to infer on tissue elasticity. Rayleigh and Love waves, which will not be further discussed, are also moving at a speed of a few m/s in biological tissues (typically < 10 m/s).

*Advanced materials*: *The speed of a compression wave is determined by the square root of the bulk elasticity modulus divided by the mass density of the tissue. The bulk modulus, or isostatic elasticity modulus, is the constant of proportionality relating the stress–strain linear behavior of a tissue deformed by a compressive movement or a compression wave in the context of this review. We recall here that ultrasound systems are considering a constant bulk modulus and a constant mass density to produce depth information on images (i.e., a constant speed of sound). However, it is the change in speed of sound at interfaces that allows observing image boundaries and tissue contrast. In other words, a constant speed of sound is required to estimate distances on the image but without speed of sound heterogeneities, no image would be produced. Notice that the concept of adiabatic compressibility can be found in the technical ultrasound literature; it corresponds to the reciprocal of the bulk modulus. It is also of value to define the concept of acoustic impedance that is the product of the speed of sound by the mass density of the tissue.*

### Ultrasound wave propagation

**Fig. 3:**
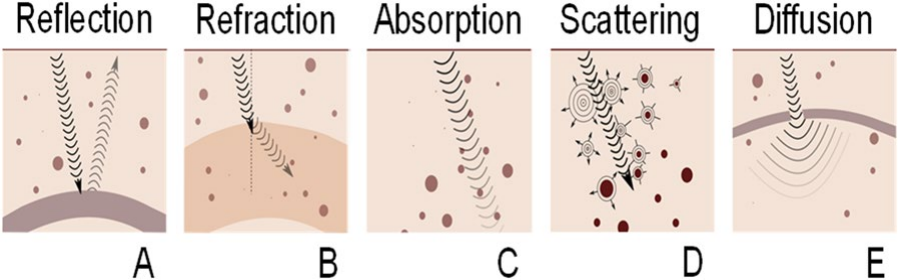
During their propagation, compression or shear waves are modified due to their interaction with the physical medium. Their direction and amplitude may change due to (**a**) reflection or (**b**) refraction at the interface of media with different acoustic impedance. The amplitude may also decrease (i.e., attenuation) due to (**c**) absorption and (**d**) scattering produced by the insonified medium. **e** Space between scatterers or within specular reflectors favor the spreading of the wave field due to diffraction. Waves received by the ultrasound transducer to produce an image are attributed to reflection and scattering

Elastic waves traveling into biological tissues experience different physical phenomena. A few concepts are relevant to explain these wave properties. Reflection, refraction, absorption, and scattering phenomena illustrated in Fig. 3, and defining ultrasound image characteristics, are determined by the speed of sound and tissue mass density, yielding the acoustic impedance. More specifically, these wave phenomena are influenced by changes in acoustic impedance along the ultrasound propagation path. All ultrasound imaging modes (Fig. 1) are affected by tissue mechanical properties (bulk or shear modulus), which are hence not specific to ultrasound elastography. Wave diffraction (Fig. 3e) characterized by the spreading of a wave around obstacles or within “holes” created by a mismatch in acoustic impedance is influenced by the relative dimension of the obstacle or hole with respect to the ultrasound wavelength *λ* (*λ* is the reciprocal of the ultrasound wave frequency).

### Image formation and point spread function

**Fig. 4:**
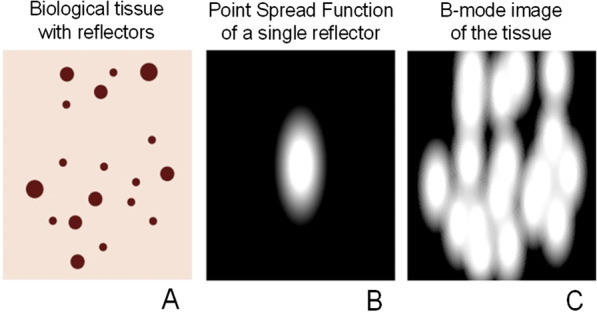
B-mode images do not provide a resolved signature of the tissue cellular content. **a** Single cells or structured connected cells forming a living tissue are providing acoustic impedance interfaces reflecting or scattering compressive waves emitted by the ultrasound probe. **b** These backward waves are detected by the same probe but are filtered by the “point spread function” of the ultrasound system. In other words, the limited resolution of ultrasound coupled with probe characteristics and scanner settings do not allow resolving single cell geometry. The “point spread function” can be interpreted as the image that would be produced by a single reflector much smaller than the acoustic wavelength but with enough acoustic impedance contrast with the ambient medium to provide a detected backward echo. **c** The B-mode image corresponds to the tissue sub-resolution characteristics filtered by the “point spread function.” The spatial distribution of reflectors and scatterers impact the final image appearance

**Fig. 5:**
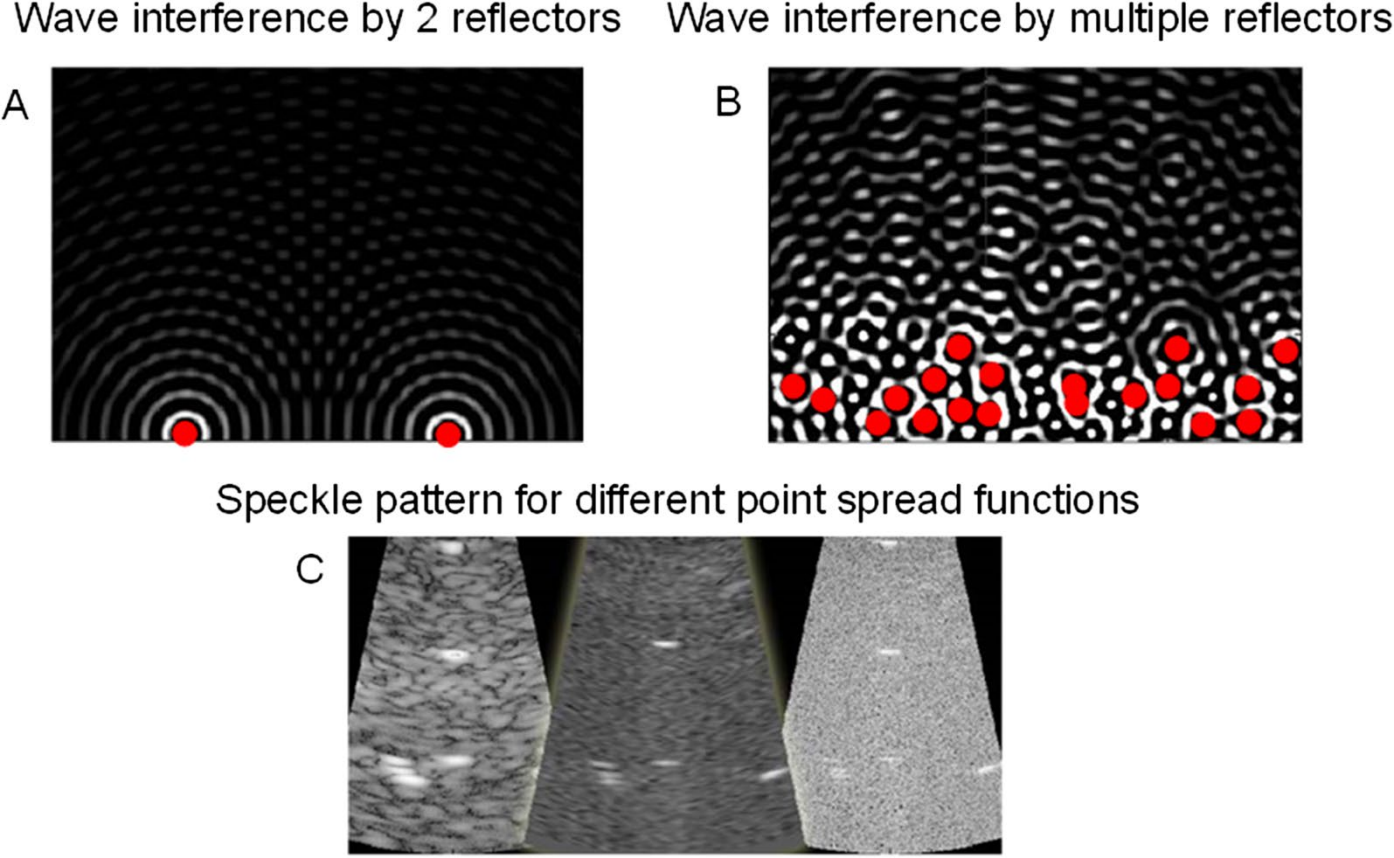
**a** Constructive and destructive wave interference patterns when forward echoes sent by a transducer (on the top of the image) are reflected or scattered by 2 particles located at the same depth. **b** Wave interference patterns when reflectors or scatterers are distributed in space. **c** B-mode images with different speckle patterns obtained from same reflectors or scatterers distributed in space, but with different point spread functions at lower to higher frequencies from left to right. The terminology “reflector” is generic here but formally a reflector has a dimension larger than the acoustic wavelength, whereas a scatterer has a dimension much smaller than the wavelength

As illustrated in Fig. 4, the speckle in B-mode images actually provide a signature of the tissue microstructural cellular content, but at a resolution determined by the system’s “point spread function” (PSF), which acts as a “blurring convolution filter.” The shape of the PSF is determined by the electrical signal transmitted to probe piezoelectric or capacitive elements, by the beam forming scheme, and by the acoustic lens characteristics. The electrical signal transmitted to the probe oscillates according to the transducer geometry and fabrication characteristics. The oscillation duration defines the transducer frequency bandwidth and axial resolution (typically 1.5 mm at 1 MHz). Its shape (e.g., sinus, square, or wavelet characteristics) modifies the frequency content of the transmitted compression wave, and its dominant frequency defines the mid-bandwidth. Beam forming also affects PSF characteristics, which is used to improve the focus laterally. An acoustic lens defining the focus in elevation (i.e., out of plane of the image) also modulates the PSF behavior and speckle characteristics. Other ultrasound system settings can be used to improve the image quality, and intervene in defining the magnitude and geometry of the PSF [19–21]. An interpretation of the PSF filtering, which characterizes ultrasound systems, is to consider the image that would be produced by a single reflector much smaller than the acoustic wavelength but with enough acoustic impedance contrast to provide a detected backward echo (Fig. 4b). As seen, that reflector cannot be resolved due to this filtering effect. By considering all reflectors depicted in Fig. 4a, the PSF contributes to the speckle pattern observed on B-mode images (this is known as the convolution effect mentioned above, Fig. 4c). The “reflector” terminology used in Figs. 4 and 5 is generic and is referring to reflection or scattered wave phenomena produced by an object.

### Constructive and destructive wave interferences

**Fig. 6:**
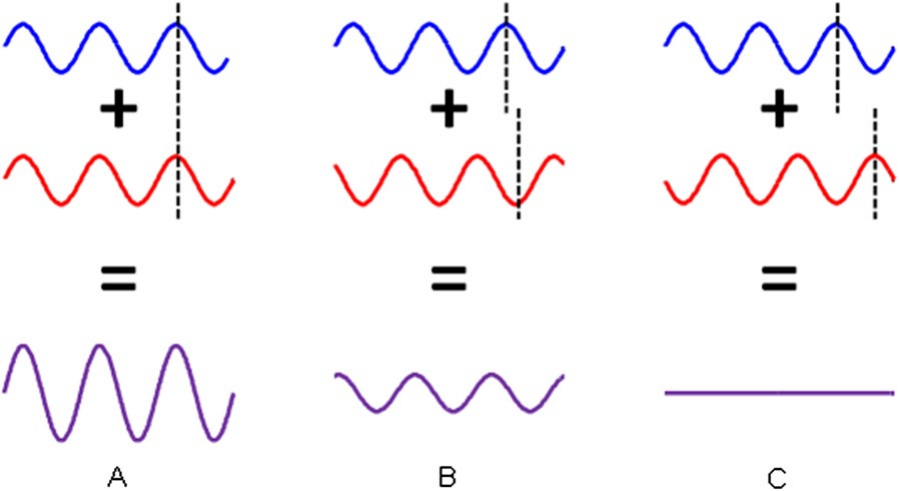
Illustration of the concept of constructive and destructive wave interferences. **a** Constructive wave interference occurs when both interfering waves (in blue and red) are in phase (i.e., aligned as indicated by the dashed line); the resulting wave (in purple) has doubled its amplitude. **b** Partial destructive wave interference observed when both interfering waves are phase delayed (as indicated by nonmatching dashed lines); in that case, the resulting wave (in purple) has a lower amplitude than both interfering waves (in blue and red). **c** A complete destructive wave interference occurring when both interfering waves are phase shifted to obtain a maximum when the other wave has reached its minimum (as indicated by dashed lines); in this case, the resulting wave (in purple) has zero amplitude

When an ultrasound wave interacts with densely packed tissue constituents, constructive and destructive wave interferences are generated: the number and spatial positioning of reflectors are also key determinants of the speckle image characteristics and are a source of the sub-resolution information gathered by QUS backscatter imaging biomarkers. The previous explanation on image formation is oversimplified because a given reflector does not lead to a specific speckle defined by the PSF (as in the case of Fig. 4c). When multiple reflectors are present (Fig. 5a, b, we consider here weak scatterers - i.e., small acoustic impedence contrasts - thus multiple reflections between scatterers are negligable), wave interferences are observed and this is also contributing to the speckle pattern characteristics. The PSF and wave interferences are modulating image characteristics. As illustrated in Fig. 5c, a given tissue with specific positioning of reflectors that is imaged with different scanners or system settings leading to different PSFs would result in quite different B-mode image appearance. The concept of constructive and destructive wave interferences is further illustrated in Fig. 6. If one considers two reflectors producing echoes with different delays among them due to different spatial positioning, the summation of these waves contributing to the final image pixel can be quite different according to the importance and value of this delay. If two echoes are in phase (i.e., no delay between them), then a constructive interference with a doubled final amplitude is observed. If two echoes are out of phase (i.e., with a phase shift of 180 degrees), then the wave interference will produce a null echo. This is typical of black pixels observed on B-mode images when an underlying tissue is expected. Intermediate conditions with phase delays between 0 and 180 degrees lead to different levels of wave interferences.

*Advanced materials*: *Intuitively, one may think that more reflectors or scatterers present in a given ROI would produce brighter images (or higher backscatter in the context of QUS), but this is not true because of wave interferences. Experimental reports using flowing red blood cells or cellulose particles embedded in a gel phantom at different volume concentrations revealed a linear relation between the backscatter intensity and the number of particles up to a volume fraction of approximately 6%, followed by a peak around 13–20% volume fractions, and a reduction in backscatter at higher number densities of particles (i.e., hematocrit in the case of blood)* [22, 23]. *Destructive wave interferences explain the reduction of the echo magnitude as the number of particles is increased beyond typically 13–20% volume fractions. This observation might be of value to interpret blood backscatter in anemia, or liver backscatter at different lipid vesicular concentrations in steatosis* [24].

### RF mode at the origin of all ultrasound imaging modalities

All imaging modes in ultrasound start from radiofrequency (RF) data processing. RF images correspond to the summation of echoes received by each element of a transducer following beam forming to improve image resolution. As indicated in Fig. 1, imaging modes are specifically processing RF data, in-phase and quadrature (I/Q) demodulated data, or simply B-mode images, as in the case of speckle tracking for cardiac strain imaging [25]. Following beam forming, a B-mode image is obtained from side-by-side RF lines (usually with interpolation) that are processed to obtain I/Q and then envelop detected echoes (i.e., a B-mode echo). The Doppler mode is based on the processing of the demodulated I/Q data to retrieve the phase shift between emitted and received echoes to apply the Doppler equation for assessing blood velocities [26]. In strain or shear wave elastography, RF data processing is privileged to improve tissue movement detection accuracy, but I/Q or B-mode data processing can be used for some algorithms [27]. I/Q data are advantageous to reduce sampling rate and data transfer load, whereas B-mode processing is of value because of the wide access to clinical B-mode image sequences.

Notice that 15–20 years ago, a few ultrasound manufacturers started providing access to RF data for research purpose (e.g., Ultrasonix Medical Corporation, Verasonics, Visualsonics, Esaote, Terason). Today, most major clinical ultrasound manufacturers can provide RF or I/Q access in addition to clinical imaging packages (e.g., Siemens Healthineers, General Electrics Healthcare, Canon Medical Systems, Samsung Healthcare, Supersonic Imagine—Hologic, etc.). Research agreements may, however, still be required to have RF access with some companies. Acquiring RF data had a major impact because it provided academic scientists the possibility of developing technical innovations with state-of-the-art ultrasound scanners.

### RF, I/Q and B-mode data formats

**Fig. 7:**
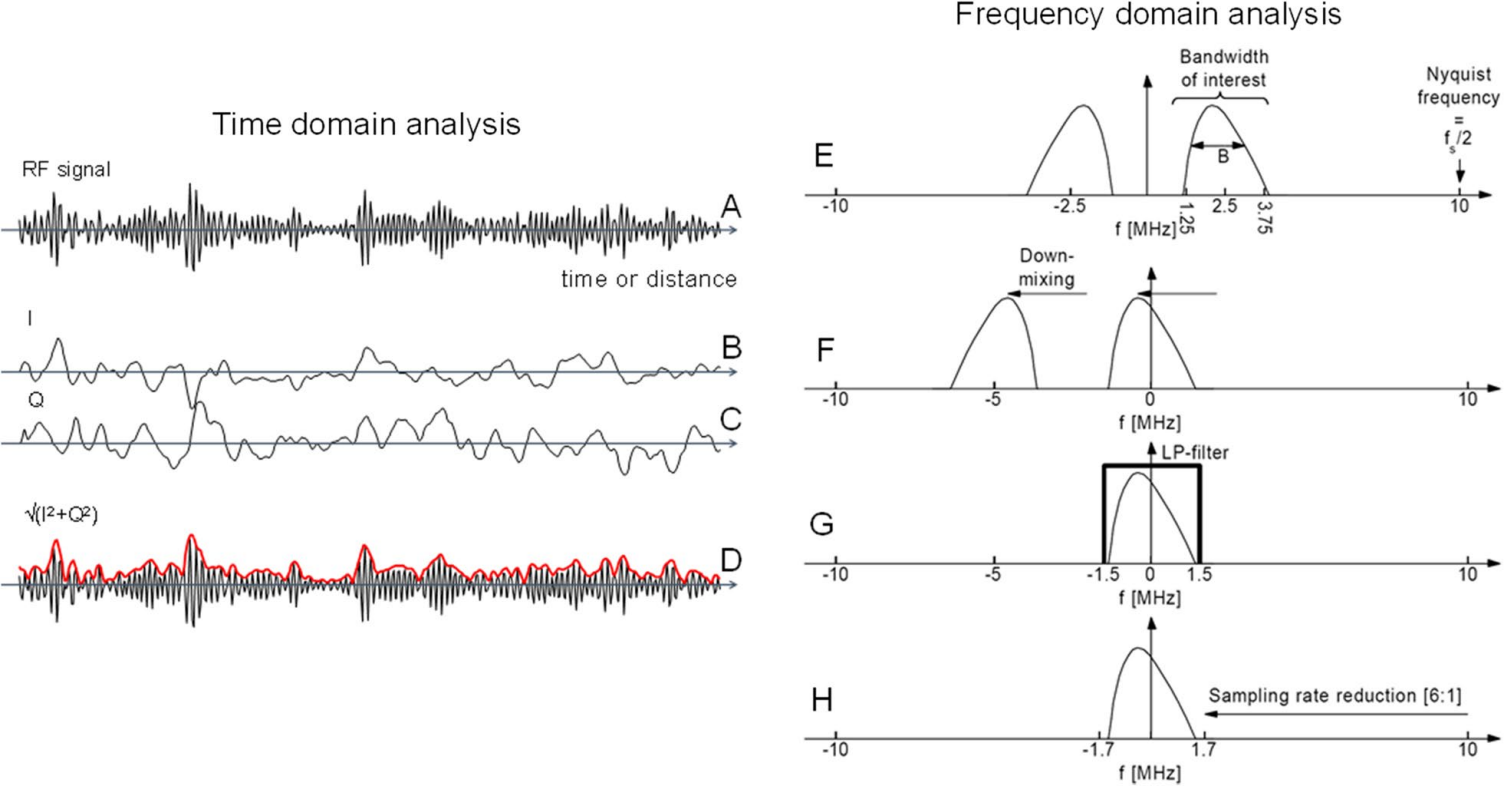
Time-domain (**a**–**d**) and frequency-domain (**e**–**h**) representation of ultrasound echoes to illustrate the difference between radiofrequency (RF), in-phase (I), and quadrature (Q) datasets. **a** The radiofrequency signal corresponds to the ultrasound echo at a given scan line on an image. **b** The demodulated in-phase and (**c**) quadrature components are obtained through signal processing implying multiplication of the RF signal by cosine (I) and sine (Q) functions at the center frequency of the probe, or of the transmitted wave. **d** The envelope detected echo used to produce a B-mode image by mapping the magnitude (red envelope) as a function of depth in gray scale is obtained as the square root of I^2^ plus Q^2^. *Advanced materials*: ***e*** *Frequency-domain representation of the RF signal, where the bandwidth of interest is governed by the ultrasound transducer characteristics. The Nyquist frequency is the minimum sampling frequency of the temporal signal required to avoid aliasing (i.e., an undersampling of the echo resulting in a wrong representation of the signal). Here a 2.5 MHz center frequency probe is considered with a bandwidth covering the frequency range of 1.25–3.75 MHz*. ***f*** *I/Q demodulation in the frequency domain (one needs to know that the Fourier transform of a temporal signal used to obtain its power spectrum results in a real—positive frequency, and imaginary—negative frequency display since it is a complex number representation)*. ***g*** *The demodulation is followed by the use of a low-pass (LP) filter to keep frequency contents corresponding to the original main positive spectrum of the RF signal. By applying an inverse Fourier transformation on the final spectral representation, one obtains the temporal I and Q complex signals*. ***h*** *For the example of this figure, the demodulation allowed reducing the sampling rate by a factor of 6*. Adapted from [28]

Figure 7 illustrates the difference between RF, I/Q, and B-mode datasets (adapted from [28]). The left panel shows time-domain processing, and the right panel presents the corresponding spectral content. The time-domain RF signal contains detected reflected and scattered echoes returning to the transducer. It is understood that constructive and destructive wave phenomena contribute to the RF signal signature. Since transmitted compression waves are selected to provide frequency contents covering the whole bandwidth of the transducer (typically by using a short transient electrical signal), and considering that frequency characteristics are modified by frequency-dependent phenomena, such as attenuation and backscatter, the received compression waves can be seen as a “filtered” representation of transmitted echoes. Once detected by the transducer in analogic form (i.e., as a time-varying voltage), analog-to-digital converters are used to obtain a numerical representation for further processing. As indicated in the top right panel of Fig. 7, the frequency content of the RF signal is in the MHz range and corresponds to the transducer bandwidth (e.g., 1.25 to 3.75 MHz for this example that might correspond to an abdominal or cardiac probe). Time-domain processing of the RF signal provides the low-frequency I/Q representation of received echoes with corresponding spectral description on the right panel. Following envelope detection (red line on the left panel) made by processing time-domain or frequency-domain echoes, its magnitude is mapped in gray levels to represent B-mode speckle at a given lateral (i.e., a given RF scan line) and depth (i.e., a given time) positions to produce an image.

### Introduction to QUS imaging

**Fig. 8:**
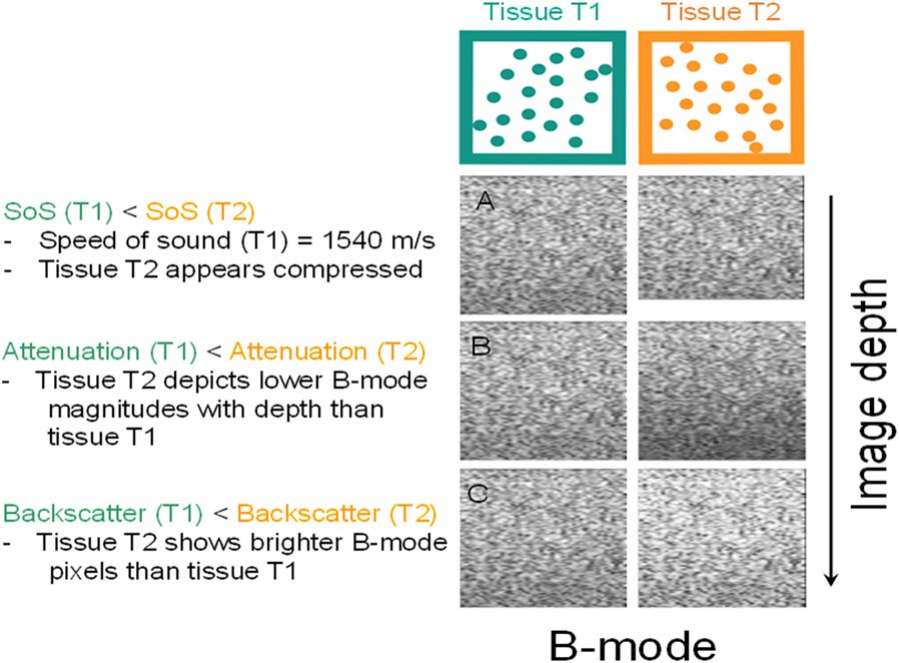
Simple interpretation of QUS imaging modes. Tissues T1 and T2 when interacting with a compression wave emitted by the transducer are characterized by different QUS properties evaluated by the speed of sound (SoS), attenuation, and backscatter coefficient measures. The interpretation of QUS parameters in this example is based on the B-mode representation of the tissue. **a** Compared with a reference tissue T1 that has a speed of sound of 1540 m/s, tissue T2 that has a higher speed of sound would appear compressed because distances on clinical ultrasound systems are measured with the assumption that all tissues behave with an SoS = 1540 m/s. **b** Tissue T2 that has a higher attenuation would appear hypoechoic in deeper locations. **c** Tissue T2 that has a higher backscatter would appear hyperechoic on B-mode imaging

Figure 8 provides an intuitive representation of speed of sound, attenuation, and backscatter measurements. Most algorithms and methods developed to provide these QUS biomarkers are based on RF data processing. RF data are more suited to track wave motion required for speed of sound imaging, and they contain spectral information used to describe attenuation and backscatter measures as a function of frequency. Since QUS imaging provides sub-resolution information relying on the cellular content and structure of a tissue, it could also be angular dependent when the measure is done on anisotropic tissues, such as muscles or tendons having fibers organized with privileged orientations. Examples of parametric images of these biomarkers are given in specific sections describing how these imaging modes are obtained.

## Speed of sound imaging

### Concept

Biological tissues are characterized by different speed of sound (SoS) providing acoustic impedance contrast.

### Units

SoS is expressed in meters per second (m/s).

### Range

Reported values measured using experimental ultrasound devices and excised soft tissues are typically varying from 1400 to 1700 m/s at body temperature [1]. SoS is lower in fatty tissues and higher in muscles and tendons, and it changes according to the pathological state of the tissue [29]. Most reports on SoS are based on technical experimental setups; tabulated values obtained using clinical implementation of the methods are scarce.

### Measurement methods

Original efforts in this field aimed at improving the quality of B-mode images corrupted by phase aberrations produced by varying SoS along the acoustic wave propagation path [30–33]. As discussed earlier (in advanced materials), a heterogeneous tissue with varying bulk moduli and mass densities can locally modify the SoS, and consequently the depth at which each component of the tissue would be represented. For example, the boundary of an organ with spatially varying SoS might be displayed at different depths due to compression wave phase aberrations, potentially affecting the quality of the diagnosis. The objective of phase aberration correction methods is to adjust locally the SoS to produce clearer images with less blurring. Technical efforts made in the field of phase aberration corrections constitute the framework of contemporary SoS measurement and imaging methods in the QUS field.

*Advanced materials*: *Phase aberration correction methods led to the development of focusing strategies relying on the estimation of the mean SoS between the face of the transducer and the focal depth of interest. Alternatively, an image processing approach also aiming at improving beam focusing was proposed* [34]. *In the latter report, the mathematical deconvolution operator was used to retrieve a restored image produced by considering an ultrasound system PSF having different mean SoS values. A focusing approach based on the estimation of SoS along the wave propagation path was specifically introduced for the purpose of providing a tissue characterization signature in* [35]. *Conceptually, since a mean estimate along the ultrasound beam is obtained with these abovementioned methods* [34, 35], *no local measure within a given ROI of an organ having inhomogeneous SoS can be produced. Current methods allowing local assessments or images within an ROI are based on spatial coherence* [36] *and image compounding* [37] *approaches*.

### Illustration

**Fig. 9:**
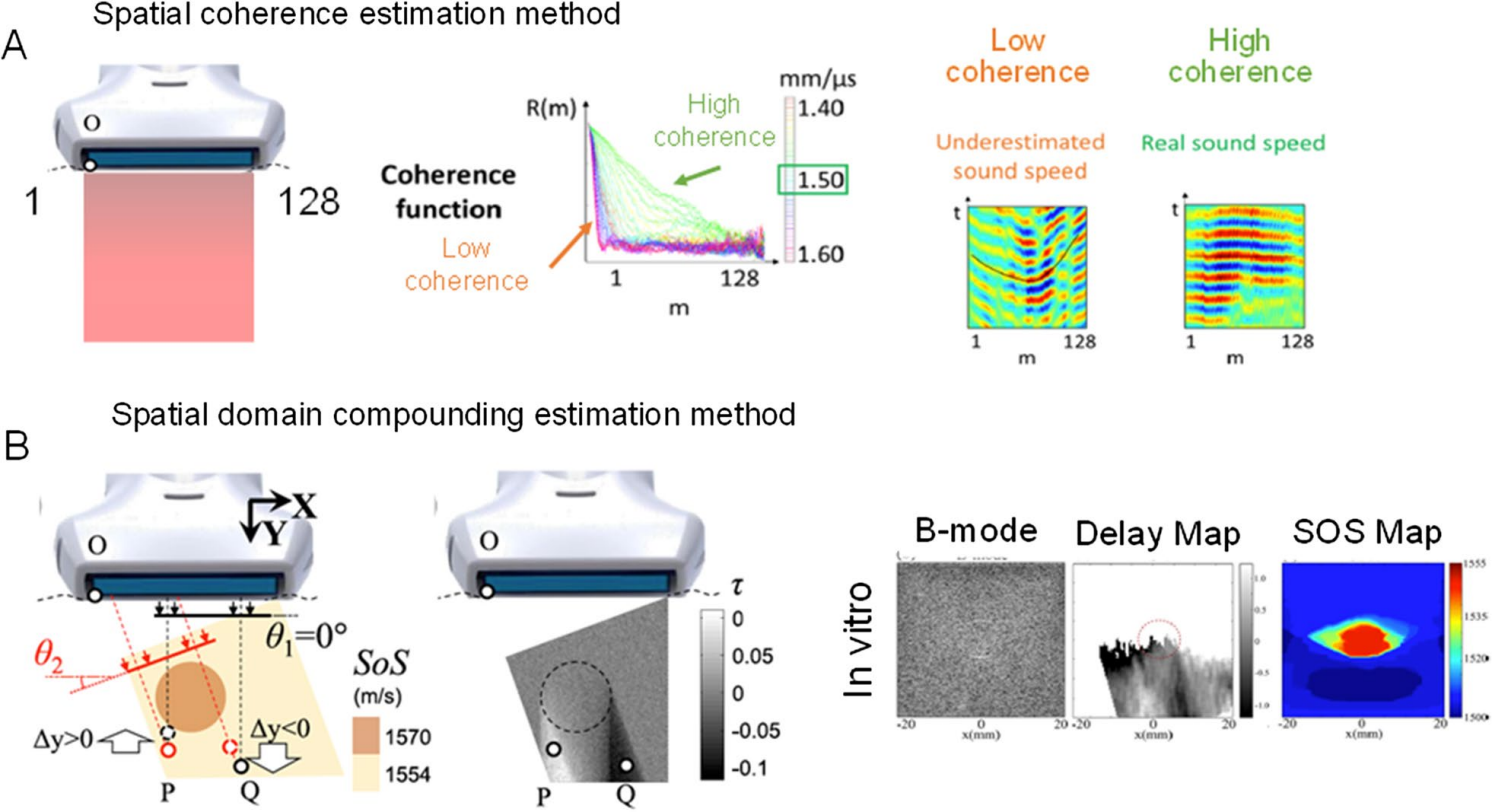
Schematic representation of current local speed of sound (SoS) algorithms. **a** Local speed of sound assessed using the spatial coherence method provides a global value in m/s without a parametric image. A tissue with a low coherence *R*(*m*) among transducer elements location (*m* = 1–128 on this example) would underestimate the speed of sound, whereas a tissue with high coherence would provide the real speed of sound. **b** Local speed of sound determined with the spatial domain compounding approach provides a parametric map of the speed of sound. Compounding is obtained by insonifying the tissue at different angles *θ*. See [37] for specific definitions of parameters used in this graph. Figure modified, adapted, and reproduced with permission from [36] and [37]

A schematic representation of the current state-of-the-art spatial coherence [36] and image compounding [37] methods developed for local SoS assessments is illustrated in Fig. 9. Another recent strategy was proposed for producing local SoS maps based on a prior estimate of the mean SoS along the wave propagation path [38].

### Clinical example(s)

**Fig. 10:**
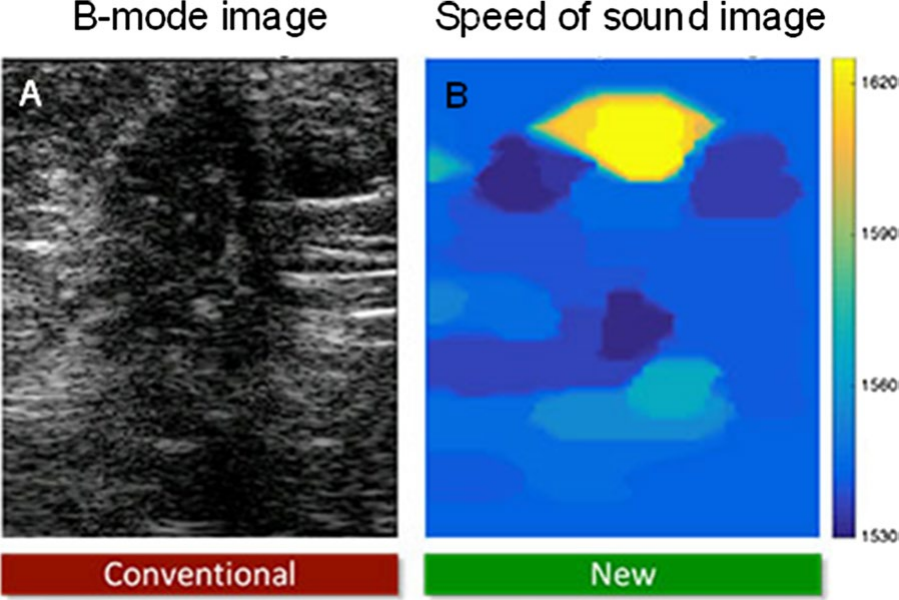
**a** B-mode image of an 88-year-old woman with a breast cancer. **b** Speed of sound map computed within the selected region of interest considered for B-mode imaging. Figure provided by Orcun Goksel from the Swiss Federal Institute of Technology, Zurich, Switzerland. Related works can be found in [37]

A few ultrasound manufacturers are today offering SoS capability for tissue characterization by providing a mean value within a selected ROI (no image), as in [39]. Imaging local heterogeneous SoS pixel values within an ROI might become available soon to clinicians; an example of such capability in the context of breast cancer imaging is given in [37], see Fig. 10 (image compounding method). It is anticipated that multiple organ diagnosis based on SoS images might spread once manufacturers will release such imaging packages. SoS measurement methods and instruments were also proposed in the context of QUS imaging using tomographic reconstructions [40–43].

## Attenuation imaging

### Concept

Ultrasound attenuation refers to the loss of mechanical energy as an acoustic wave propagates in soft tissues [4]. In addition to compression wave absorption (i.e., transfer of energy into heat), wave reflection, refraction, diffraction, and scattering are also contributing to the diminution of the wave amplitude with distance by redirecting acoustic energy away from the field of view of the transducer. The attenuation can be qualitatively appreciated by clinicians performing a standard B-mode examination. The magnitude of the echo within the image is reduced by attenuation, and loss of structural details over depth and shadowing can be observed. Thus, attenuation may be considered as an imaging artifact or as a specific feature of the tissue with diagnostic value. For a proper understanding of concepts described below, “total” attenuation means the attenuation between the ultrasound probe and an organ at a given depth (thus considering the contribution of all tissues along the path, e.g., the skin, fat layers, muscles, blood vessels, etc.), whereas “local” attenuation refers to the attenuation within an organ ROI to provide a tissue signature. Images might be reported using both total and local attenuation measurements. The characterization of an organ based on QUS attenuation measures or images usually refers to local attenuation assessment.

### Units

Since attenuation is the ratio of one RF magnitude to another at a different depth, it is expressed using the unit of decibel (dB) computed as 20 times the logarithm (in base 10) of this ratio. Also, because ultrasound attenuation increases with frequency, it is reported in dB/cm/MHz. If one assumes that the attenuation on a log scale varies linearly with frequency (which is not the case of all biological tissues [44]), the value at a higher frequency may be obtained by multiplying the attenuation in dB/cm/MHz by the frequency in MHz to obtain a measure in dB/cm at a given frequency (which is often reported). By assuming such a linear dependency with frequency, the relation between attenuation versus frequency is obtained by computing the slope of this relation; for this reason, it is common to report attenuation measures with the acronym “attenuation coefficient slope” (ACS).

### Range

Experimental measures of the ultrasound attenuation coefficient at body temperature using through transmission (i.e., 2 transducers located on both sides of the tissue) or reflection (i.e., one transducer for emission and reception) instrumentations vary from 0.01 dB/cm/MHz in blood to 4 dB/cm/MHz in muscles [1, 45]. These values correspond to extremes reported in technical reports based on experimental setups and ex vivo tissue samples.

### Measurement methods

**Fig. 11:**
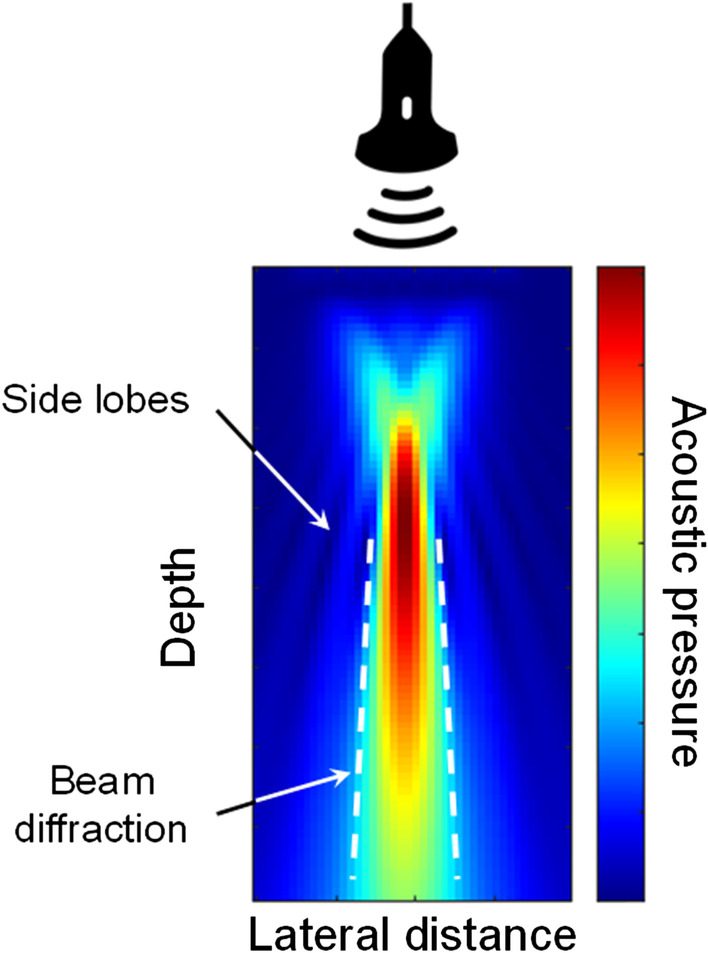
Typical diffraction pattern of an ultrasound probe and presence of side lobes associated with the beam forming reconstruction method, and finite dimension of the transducer aperture (typically determined by the number of transducer elements used at emission and reception). Notice that diffraction and side lobe characteristics are defining the point spread function property schematized in Fig. 4. This example illustrates the acoustic pressure distribution of a typical ultrasound beam produced by an array transducer. Specific beam forming strategies are used to reduce side lobes and multiple focuses improve the uniformity of the acoustic pressure distribution. Nevertheless, wave diffraction occurs and should be compensated to achieve reliable QUS measurements. Because the magnitude of compression waves is not uniform in the lateral (*x*-axis) and axial (*y*-axis or depth) directions, calibration with a reference phantom is required to compensate for spatial changes

There are two main methods for estimating the local attenuation coefficient slope, i.e., the attenuation in dB/cm/MHz within a pre-specified ROI: the spectral difference [46, 47] and the spectral shift [48] methods. With the former approach, the attenuation coefficient slope is estimated from the reduction of the echo signal power with depth, whereas for the latter, it is deduced from the downshift in center frequency of the backscatter echo with depth due to the frequency-dependent attenuation. Variants of these strategies were proposed, namely the spectral log difference method [49] and a hybrid method [50]. These algorithms are assuming that the scattering properties (i.e., the backscatter coefficient) of the organ are unchanged over the depth range of the ROI. Moreover, a calibration method is required to compensate for the compression wave diffraction confounder of the transducer, which also reduces the echo magnitude with depth. As illustrated in Fig. 11, a compression wave emitted by an ultrasound transducer does not have uniform magnitude laterally and axially following beam forming; consequently, a calibration is required to compensate for spatial changes in magnitude.

To perform the calibration, echo signals from a reference phantom whose attenuation is known must be obtained using the same equipment and system settings as the clinical examination. Such calibration phantoms are available commercially (e.g., Sun Nuclear or CIRS). Because of the often assumed linear frequency dependence of the logarithm representation of local attenuation, such calibration phantoms may be used for the whole frequency bandwidth of clinical array transducers. The ratio of power spectra from the tissue and reference phantom within an ROI yields the attenuation coefficient of the scanned organ at the frequency and depth of interest. System effects such as diffraction, beam forming, and transmitted acoustic power are accounted for by applying the reference phantom method [51]. Tissue diffraction (Fig. 3e) is a QUS signature captured by attenuation measurements. Gain, time-gain compensation, and image filtering settings on clinical scanners, which affect B-mode images, usually do not impact RF images and thus the computation of attenuation.

### Illustration

**Fig. 12:**
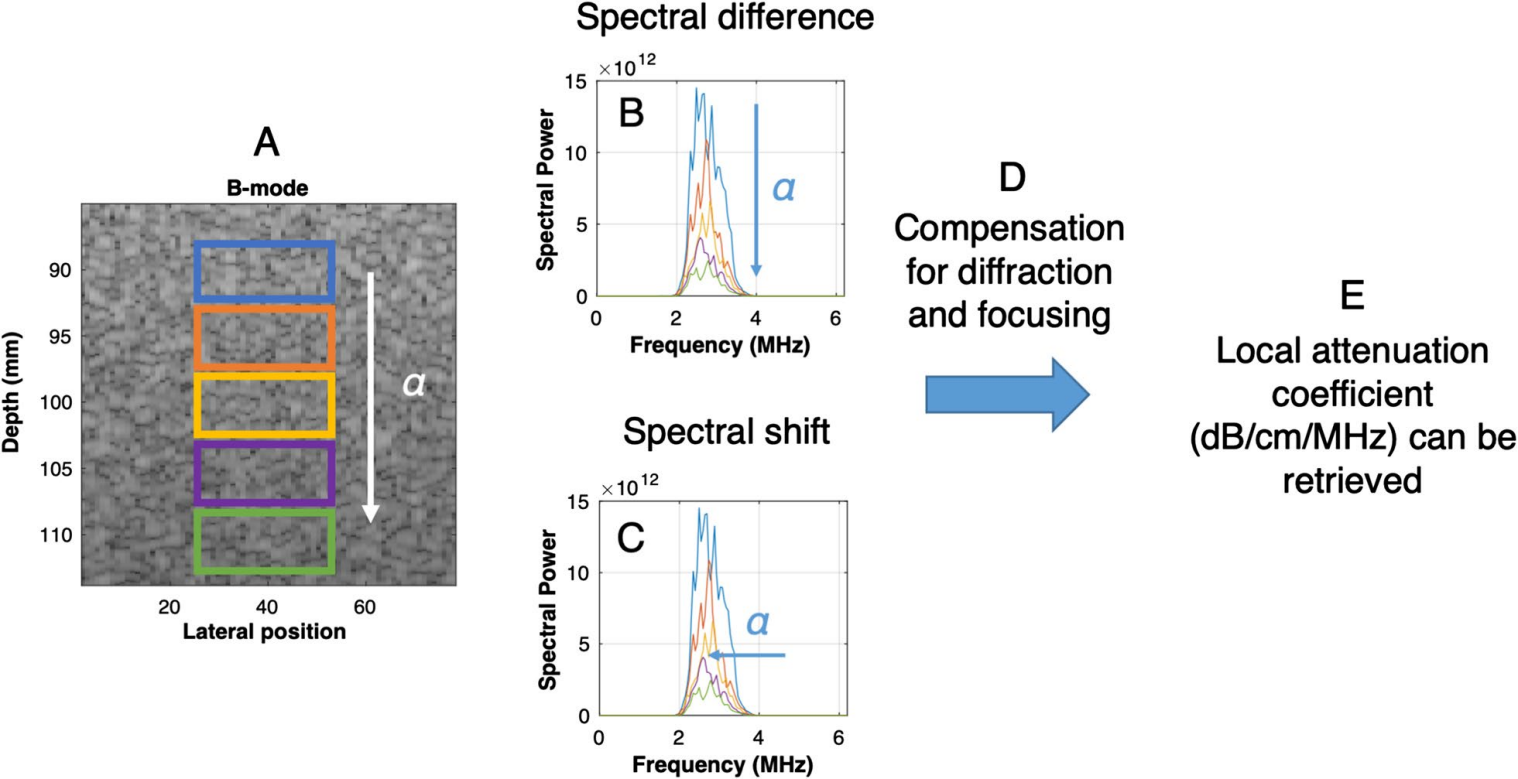
Schematic representation of contemporary local attenuation algorithms. **a** A region of interest within a B-mode image is discretized into rectangular measurement windows to locally estimate the attenuation coefficient *α*. **b** Spectral difference method, (**c**) spectral shift method, (**d**) compensation for diffraction and ultrasound system focusing using the reference phantom method, and (**e**) estimation and display of the local attenuation coefficient

A schematic illustration of the contemporary spectral difference and spectral shift methods used to assess local attenuation is given in Fig. 12. It displays an ROI within a B-mode image with measurement windows used for computation, along with a simplified representation of the phantom calibration approach.

### Clinical example(s)

**Fig. 13:**
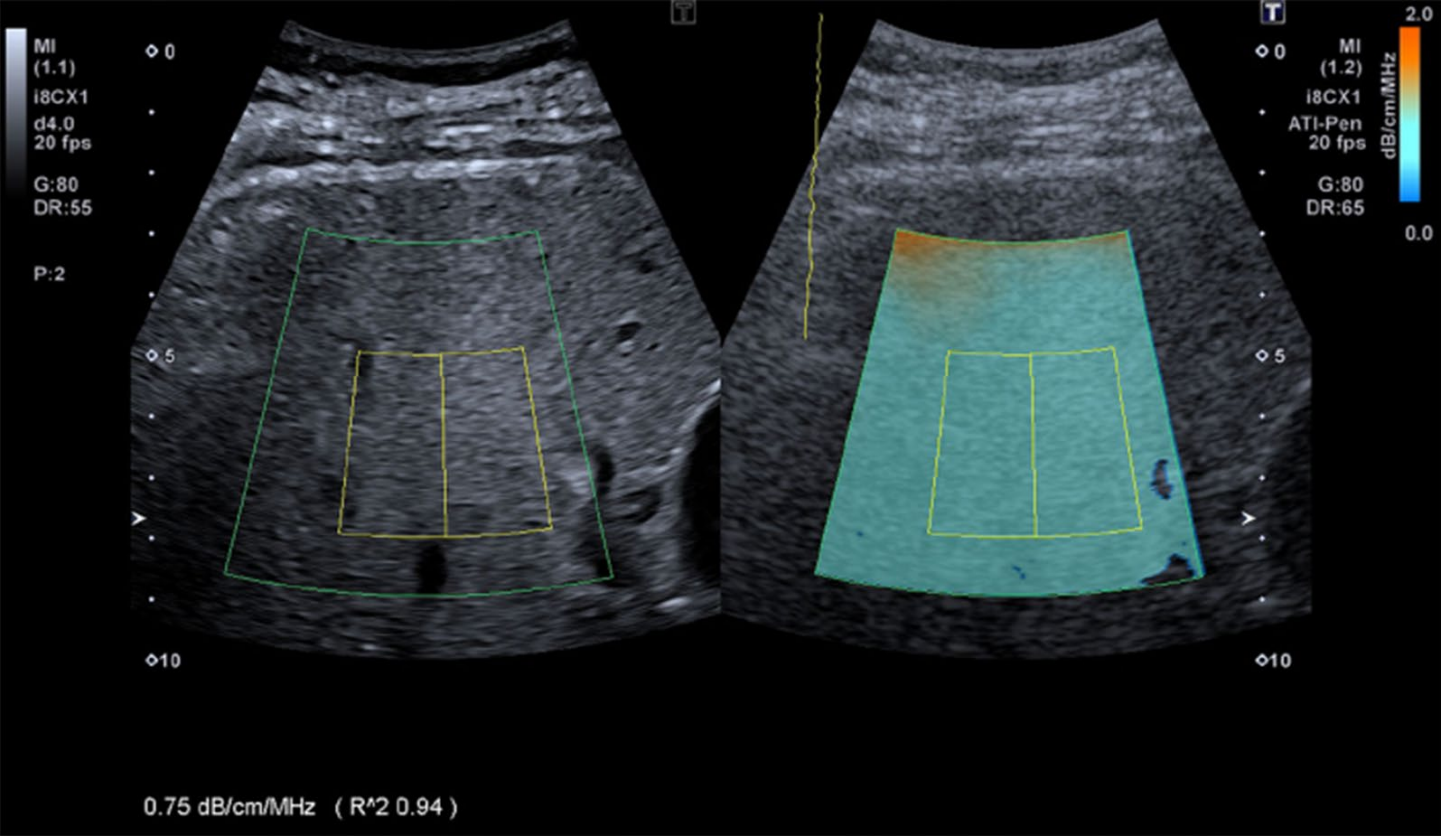
Local attenuation map of a 55-year-old man with nonalcoholic fatty liver disease reporting a mean attenuation within the selected ROI of 0.75 dB/cm/MHz, indicating a stage 1 to 3 liver steatosis. Reproduced with permission from [54]

A few ultrasound manufacturers are offering real-time attenuation images on their scanners [52–55], or values computed within an ROI with no imaging capability [56–58]. Other manufacturers reported post-processed RF datasets on remote computers with no capability of real-time attenuation imaging [59–62]. To compensate for ultrasound system wave diffraction, ultrasound beam focusing, and other system settings, most manufacturers opted for an embedded calibration (from either a training dataset or a preset reference phantom measurement). Figure 13 shows an example of local attenuation imaging implemented on a commercial clinical system for the assessment of liver steatosis.

## Backscatter imaging

### Concept

Backscatter imaging refers to the analysis of echoes received by the ultrasound transducer due to compression wave reflection and scattering, which are modulated in magnitude by constructive and destructive wave interferences produced by the tissue microstructure. A backscatter image therefore reflects the tissue microstructure, but varies with ultrasound frequency. For assessing the backscatter tissue property, a compensation for compression wave attenuation along the propagation path should be considered to get a signature of the intrinsic distribution in acoustic impedance inhomogenities of the tissue producing backscatter echoes. This is accomplished by considering the total attenuation along the path between the emitted compression wave at the surface of the skin to the ROI within the insonified organ [63, 64].

### Units

**Fig. 14:**
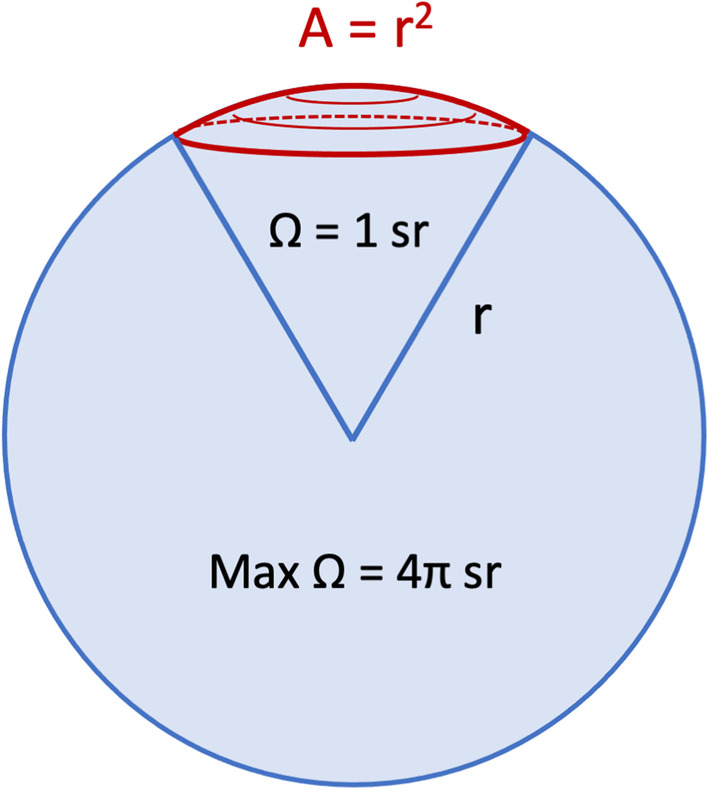
Illustration of the steradian dimensionless unit used to define the backscatter coefficient. The solid three-dimensional angle Ω corresponds to the area *A* divided by the radius squared of the sphere

The most recognized approach to describe backscatter in the field of QUS imaging is to use the magnitude squared and frequency dependency of RF echoes received by the transducer to compute the backscatter coefficient (BSC) [65]. By definition, the BSC at a given frequency corresponds to the time-averaged scattered intensity in the backward direction per unit solid angle per unit volume normalized by the time-averaged incident wave intensity (cm^−1^ Sr^−1^) [4]. Practical implementation may require sampling at different probe positions and/or orientations to get spatial averaging of the tissue BSC. Units are per cm and per steradian, where the latter is a dimensionless measure referring to the ratio of the area subtended by the square of its distance from the center to that distance squared, as illustrated in Fig. 14. In this figure, the origin of the subtended angle may correspond to the central position of a scatterer within a specific ROI comprising multiple acoustic scatterers.

*Advanced materials*: *By definition, BSC < 1 (no backscatter amplification) unless hypothetically a specific ROI would contain scatterers with a reflection coefficient of 1 (i.e., no loss in energy between transmitted and reflected waves, which would correspond to a huge contrast in acoustic impedance), and these scatterers would need to be organized spatially in such a way that mainly constructive wave interferences would be present. Such conditions are not expected for biological soft tissues so that BSC is a fractional measure*.

### Range

As for previous SoS and local attenuation values, BSC measurements have been experimental and mainly based on laboratory instruments; approximate extreme values are given here for soft biological tissues. Reported values for porcine whole blood at a normal hematocrit and a frequency of 7.5 MHz vary from ≈ 0.1 to 5 × 10^–3^ cm^−1^ Sr^−1^. The range of BSCs for blood at a given frequency is explained by the modulating effect of erythrocyte aggregation and flow condition [22, 66]. The mean BSC at 3 MHz of human fatty livers is ≈ 7 × 10^–3^ cm^−1^ Sr^−1^ compared with a mean value of ≈ 0.5 × 10^–3^ cm^−1^ Sr^−1^ in healthy livers [67]. At low frequencies (typically < 10 MHz), BSC has a frequency dependency varying from ≈ *f*^2^ to *f*^4^ so that values at higher frequency are much higher for a given tissue. When the frequency is further increased, oscillating behavior with BSC peaks and troughs is observed and corresponds typically to the microstructure of the tissue and mean scatterers’ size.

### Measurement methods

Similarly to local attenuation estimation requiring a reference phantom calibration method, backscatter assessment using the BSC also needs a calibration to account for ultrasound system characteristics and settings [68]. A requirement for BSC measurements is to obtain a reference BSC in cm^−1^ Sr^−1^ at a specific frequency, or at different frequencies within the bandwidth of the ultrasound transducer, to compare the RF signal from the insonified organ to that of the reference for calibration. Commercially available reference phantoms provide BSC values but usually at a single frequency. Contrary to local attenuation in dB/cm that varies almost linearly with frequency, the BSC has a more complex tissue specific frequency behavior [3, 4], as mentioned above. Consequently, additional effort is often required to calibrate the BSC of the reference phantom at a specific frequency or frequencies of interest.

As summarized by Wear et al*.* [65], the assessment of the BSC has been associated with a greater variability than SoS or attenuation measurements due to the greater variety of measurement procedures, algorithms, and mathematical formulation relying on specific scattering theories. Using a more standardized way to compute the BSC with clinical array transducers, more robust estimates have been reported in vitro [69, 70] and in vivo [71, 72]. The framework of all BSC methods relies on similar concepts [73].

### Illustration

**Fig. 15:**
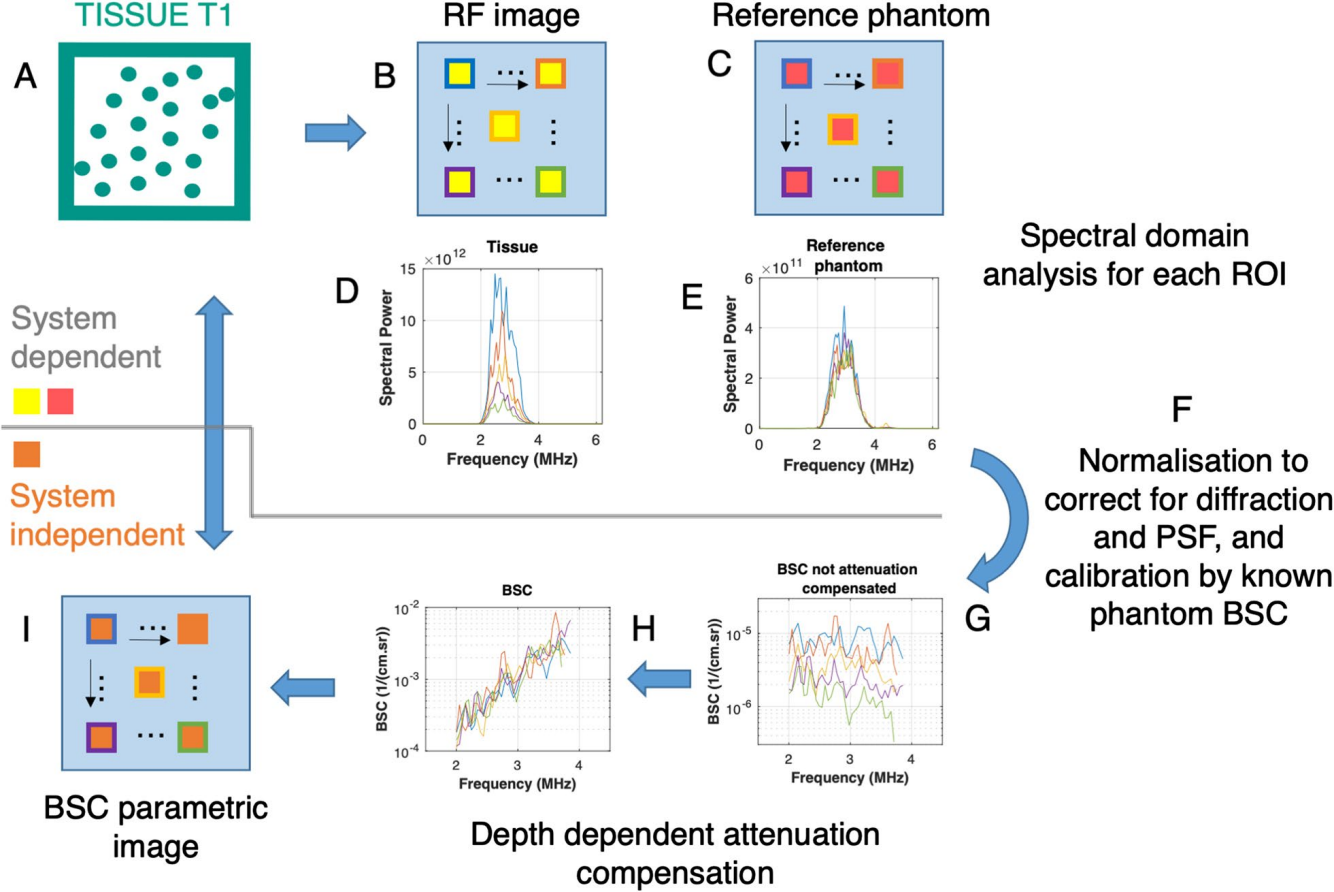
Block diagram describing processing steps required to compute the backscatter coefficient (BSC). **a** A biological tissue, containing ultrasound scatterers distributed in space, is interrogated by an ultrasound wave. **b** The interaction of the ultrasound wave with the tissue produces a backscattered RF image discretized into rectangular measurement windows. **c** Using the same ultrasound system, a reference RF image is taken on a reference phantom with a known BSC, and discretized rectangular measurement windows are distributed at the same depth and lateral positions as in the case of the tissue image. **d, e** At every location of a measurement window, power spectra are computed for both images. **f** To eliminate effects of the system point spread function (PSF) and beam diffraction (which are system dependent and not tissue dependent), a spectral ratio is computed at each location and calibrated by the known BSC of the reference phantom. **g** At this stage, the tissue BSC is still affected by total attenuation, which needs to be removed. **h** Depth-dependent attenuation is removed for each measurement window location, which yields a BSC parametric image in **i** that is system independent. Averaging over multiple acquisitions with different probe positions and/or orientations is practically required to improve robustness

Figure 15 shows requirements necessary to report calibrated and robust BSC values; measurements are typically taken in the frequency domain using Fourier transforms applied to RF signals. With a reference phantom properly calibrated to provide known BSCs within the bandwidth of the transducer, a clinical measurement of RF datasets within a specified ROI (i.e*.,* tissue T1) at a given depth in an organ is taken, followed by a second measurement on the reference phantom for the same ROI, keeping all ultrasound system settings the same. As indicated in Fig. 15, the mean RF power spectrum of the clinical measures computed over a few frames is divided by the mean RF power spectrum of the reference phantom, and then the result is multiplied by a function used to compensate for total attenuation. For this purpose, the total attenuation of the reference phantom at the measurement window depth should be known, along with an estimate of the total attenuation at the measurement window location within the imaged organ. The latter measure might be quite challenging, especially in obese patients with layered fat structures above the organ of interest [70]. For this reason, recent clinical reports assumed a constant total attenuation coefficient slope between the probe and the liver ROI for steatosis grading characterization with the BSC [74, 75]. As indicated in Fig. 15, because the BSC is computed within measurement windows of a given ROI (i.e., tissue T1) in an organ, methods used either the total attenuation up to the measurement window or the total attenuation up to the ROI added to the local attenuation up to the measurement window. One assumption in the calculation of the BSC is the postulate of a similar speed of sound for the clinical tissue sample and reference phantom, which can be a source of variability.

*Advanced materials*: *As mentioned above, a first prerequisite for BSC computation is the availability of a reference phantom with a known BSC for the calibration of diffraction and ultrasound system settings. If such phantom is not available or not calibrated at the frequency of interest or within the frequency bandwidth considered, a pre-step additional calibration is required. This is done by using another reference phantom mimicking specific theoretical scattering conditions. Notice that a few fundamental research laboratories may have the expertise to fabricate such phantoms, and to match acquired RF spectra to the appropriate theoretical scattering model. Once the pre-calibration phantom is fabricated, it is usually recommended to calibrate the reference commercial phantom for clinical use. Indeed, the lifetime of such pre-calibration phantoms is usually limited by dehydration, bacterial contamination or structural fragility, thus preventing them to be used as a reference for clinical BSC measurements*.

*The theoretical Faran model describing scattering by hard spheres is the most often used for BSC calibration* [76]; *it is appropriate to model the BSC of a low number density of small glass spheres randomly distributed within a solidified gel, which is a common fabrication process for BSC reference phantoms. The requirement of a low number density of scatterers is to avoid constructive and destructive wave interferences not supported by this theoretical model. It also avoids multiple reflections due to the use of glass spheres with a high acoustic impedance mismatch with the surrounding gel. With proper total attenuation compensation, RF spectra acquired on the pre-calibration phantom embedding scatterers of a uniform size are then compared to the Faran model to calibrate the commercially available reference phantom at frequencies of interest. This is achieved by repeating measurements on that commercial phantom whilst keeping ultrasound system settings the same*.

*Alternatively for blood BSC assessment on patients to assess specific conditions, such as systemic inflammation* [77] *or risk of deep vein thrombosis* [78], *a suspension of erythrocytes washed with saline to remove plasma proteins that are responsible for cell aggregation is a common practice for reference phantom calibration* [79]. *To avoid wave interference effects, a low 4–6% hematocrit suspension datasets are used and compared to the Percus–Yevick scattering model* [80, 81] *to obtain the reference BSC(f). An advantage of this calibration process is to keep similar flow condition between calibration and clinical measurements* [82], *since it is affecting the BSC of blood*.

### Clinical example(s)

To our knowledge, no ultrasound manufacturer has yet implemented quantitative BSC on their scanners. However, technology releases are in development [62] and are using an embedded calibration with preset reference phantom measurements. Alternatively, relative backscatter measures (or relative echogenicity) have widely been reported, as in the case of the hepatorenal index implemented on a few clinical scanners for liver steatosis characterization [83]. However, such measures depend on the instrument, organ assessed or used for normalization (e.g., kidney in the case of the hepatorenal index, or blood and artery wall adventitia in the case of atherosclerotic plaque analysis [84]).

## Investigational backscatter imaging methods

*Advanced materials*: *Modeling approaches were proposed to consider the whole bandwidth of the BSC instead of a single measurement at a given frequency. The rationale is relevant because the spectral representation of the BSC and especially its oscillating frequency behavior may provide unique description of the sub-resolution structural characterization of a tissue. Numerous reports thus described backscatter descriptive approaches to fit the behavior of BSC(f) to retrieve imaging biomarkers* [4]. *We do not aim at being exhaustive but simple metrics such as the spectral slope, mid-band fit, and spectral intercept have been widely used. For example, these metrics, which do not rely on physical modeling but on frequency fitting characteristics of BSC(f), allowed following breast cancer treatment response to chemotherapy* [85]. *By considering a Gaussian-shaped form factor of scatterers distributed randomly within an insonified tissue, physical modeling of BSC(f) allowed retrieving other diagnostic features, known as the effective scatterer diameter and effective acoustic concentration* [86]. *Images based on these backscatter metrics have been reported for in vivo tissue analyses* [87]. *Inspired by blood backscatter modeling, for which a structure factor was introduced to model constructive and destructive wave interferences attributed to the scatterers’ spatial positioning* [88], *BSC physical models considering a form factor of scatterers, and a structure factor, were proposed and used for spectral analyses of other tissues than blood* [89–91]. *The model behind these latter developments is known as the structure factor size estimator (SFSE)* [92].

## Statistical backscatter modeling

**Fig. 16:**
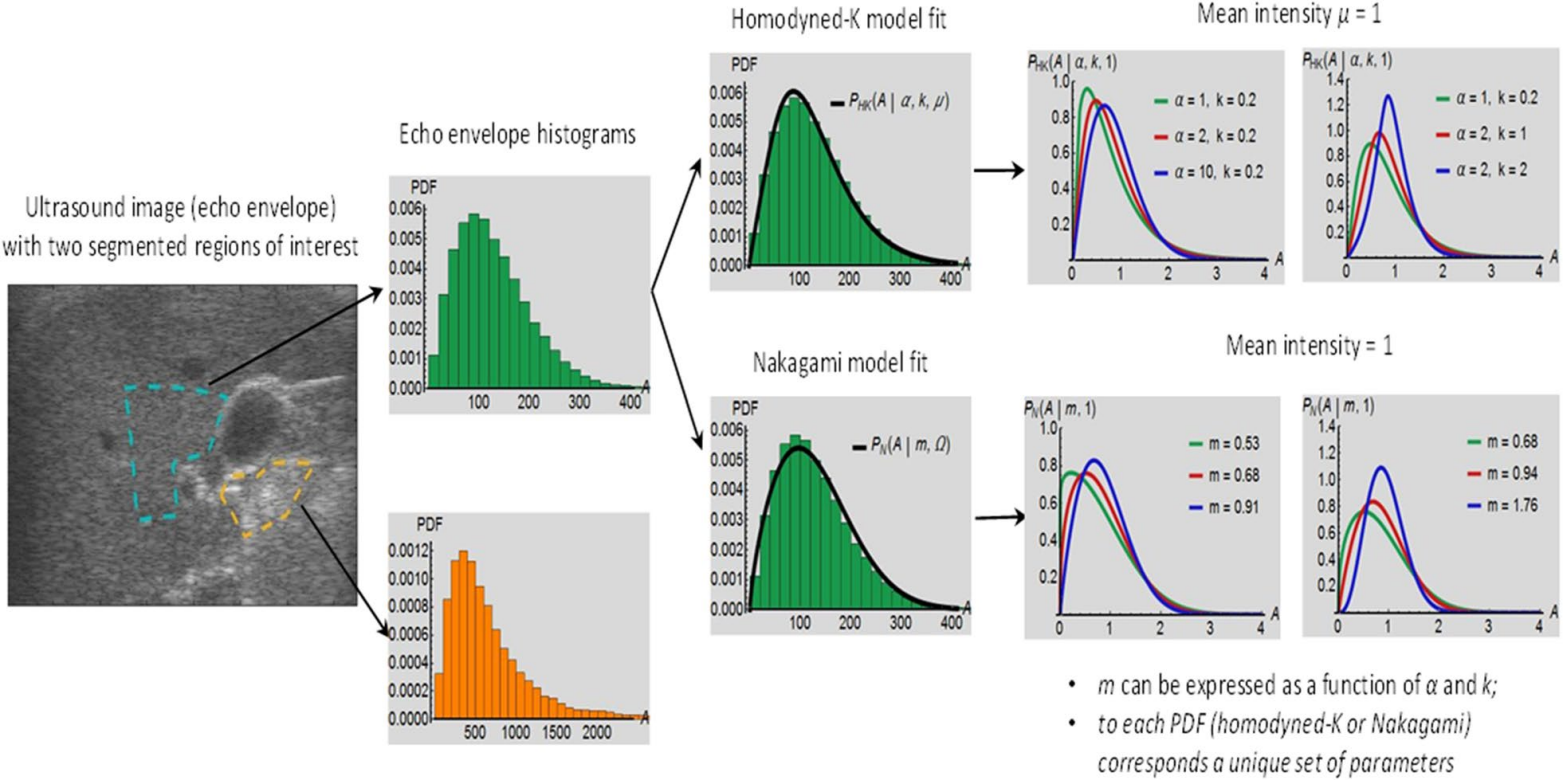
Various statistics may be computed on the echo envelope data within a region of interest. Prior to modeling with a probability density function (PDF), a histogram of the echo magnitude is computed within a specified region of interest. Two models with meaningful parameters can be used to fit the data: (1) the homodyned-*K* distribution (HDK), which is a general model with one scale parameter (the mean intensity *μ*) and two shape parameters (*α* and *k*); and (2) the Nakagami distribution, which is an approximate simpler model with one scale parameter *(Ω)* and a single shape parameter (*m*). As illustrated, changing the shape parameter(s) allow(s) modeling different forms of the image histogram corresponding to different backscatter tissue properties. *P*_*HK*_ is the PDF of the fit with the HDK model; and *P*_*N*_ is the PDF of the fit with the Nakagami model

Alternatively to the BSC, first-order statistical properties of the RF echo envelope (i.e., B-mode speckle) have been used as a signature of the tissue microstructure [93]. Pioneer works showed potential of these backscatter measures for breast and liver image analyses [94, 95]. The basic concept relies on modeling the magnitude of speckle with probability density functions. Figure 16 illustrates the concept of speckle analysis with homodyned-K (HDK) and Nakagami statistical models. Parameters used to fit the model to a histogram distribution representing the dataset can be presented in the form of color parametric images overlaid on B-mode images for diagnosis [96].

**Fig. 17:**
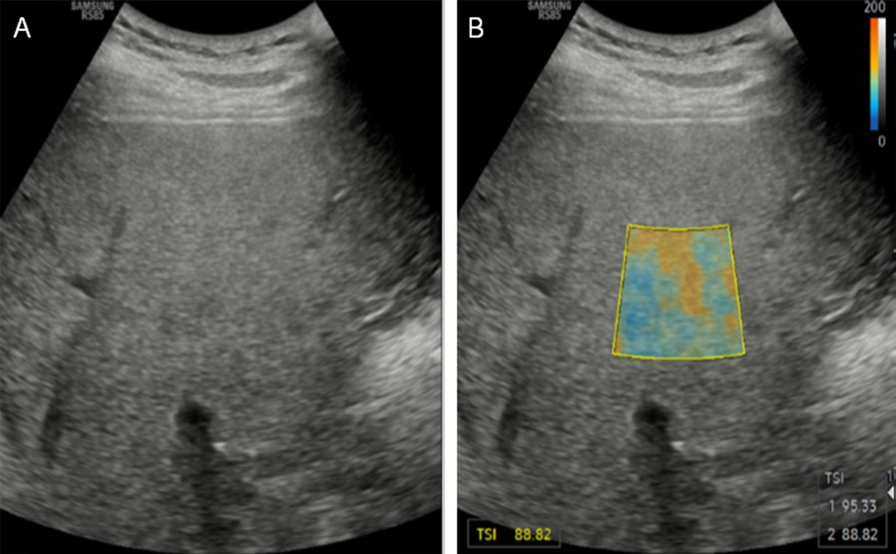
**a** B-mode image of an unidentified patient with suspected liver steatosis. **b** Tissue scattering imaging (TSI) based on Nakagami histogram modeling and post-processing. Reproduced with permission from [55]

Recently, an ultrasound manufacturer has released backscatter imaging based on Nakagami modeling [55, 97] (Fig. 17). The color map in Fig. 17 may indicate that proprietary post-processing was performed because the range does not correspond to expected values for the scale and shape parameters of this model. This is a technique that has advantages in terms of reduction of the computational complexity when compared with the BSC, but the independence to ultrasound system settings and total attenuation remains to be fully validated. Alternatively, another ultrasound manufacturer is proposing an imaging mode based on normalized variance statistics [98].

*Advanced materials:Before concluding this educational review, a last explanation is given to help appreciating the relevance of HDK or Nakagami tissue modeling for QUS imaging. Long standing developments in the field of QUS aimed at interpreting or providing structural meaning to fitted parameters obtained from statistical speckle models. This was mainly done through acoustic physics simulations or comparison of model parameters with ex vivo tissue histopathology slides. Without going into details, unifying concepts were recently made between BSC spectral modeling with the SFSE, and HDK statistical representation of backscatter echoes* [99, 100]. *The total, coherent (i.e., from spatially organized scatterers), and diffuse (i.e., from randomly distributed scatterers in space) signal powers related to HDK modeling were expressed explicitly in terms of the structure factor of the BSC SFSE model* [99]. *Also, the scatterer clustering parameter of the HDK model (α in Fig*. 16) *could be related to the packing factor of the SFSE model. We can thus conclude that BSC(f) and HDK speckle modeling (or Nakagami modeling) share common sub-resolution tissue descriptors*.

## Future directions

The field of QUS imaging is very active and recent innovations have aimed at improving image quality by using mathematical regularization algorithms [49, 101–106]. Indeed, most QUS imaging methods rely on computations made over windows that are typically larger than the PSF (5–10 times), which degrades the spatial resolution compared to B-mode imaging. Spatial filtering might be employed to reduce the window effect but regularization has the additional advantage of reducing computed outlier values appearing as background noise on QUS images. The development of phantom-free methods in QUS imaging is also a trend to pursue to improve the clinical workflow [107]. The efforts made by a few ultrasound manufacturers in this direction deserve to be acknowledged.

Artificial intelligence has started to be introduced in the field of QUS imaging to improve the tissue sub-resolution signature [108]; this may certainly play a major role in the future. Notice finally that the assessment of the total attenuation between the ultrasound probe and the tissue of interest remains a challenge for improving the robustness of BSC computation [64]. Mathematical optimization algorithms were proposed to simultaneously model the frequency dependency of the BSC with an assessment of the total attenuation [109–111]. Robustness and validation of these methods remain to be demonstrated.

A last word goes to the QIBA-PEQUS (quantitative imaging biomarkers alliance–pulse echo quantitative ultrasound) committee of the Radiological Society of North America and American Institute of Ultrasound in Medicine that should release soon guidelines on the clinical use of QUS imaging methods for liver steatosis assessment. A similar effort is conducted by the World Federation of Ultrasound in Medicine and Biology [112].

## Conclusion

QUS has been investigated for more than 50 years and is undergoing a resurgence, particularly for liver steatosis applications. The recent introduction of dedicated instruments using QUS techniques and implementation of speed of sound, local attenuation, and backscatter statistical packages on clinical scanners should contribute to their clinical adoption. Backscatter coefficient packages may also become available soon on clinical scanners. This educational review emphasized the fact that the ultrasound image texture is determined by the relationship between the spatial distribution of acoustic scatterers, their acoustic impedance discrepancy with respect to surrounding tissues, and characteristics of the ultrasound source. Acoustic scatterers in biological tissues are neither randomly nor perfectly ordered, and are generally present at a high number density, which has an impact on wave propagation. The ultrasound image texture is determined by constructive and destructive wave interferences affected by the density of scatterers, and their distribution in space. QUS methods provide a signature of tissues’ physical property, which may be represented as parametric images for diagnosis. The introduction of QUS techniques in clinical care should provide additional diagnostic tools to clinicians.

## Data Availability

No data or material is available for public distribution.

## Abbreviations

ACS: Attenuation coefficient slope
BSC: Backscatter coefficient
HDK: Homodyned-K
I/Q: In-phase and quadrature datasets
PDF: Probability density function
PSF: Point spread function
QUS: Quantitative ultrasound
RF: Radiofrequency
ROI: Region of interest
SFSE: Structure factor size estimator
SoS: Speed of sound
UTC: Ultrasound tissue characterization
